# Characterization of vaccinia virus A12L protein proteolysis and its participation in virus assembly

**DOI:** 10.1186/1743-422X-4-78

**Published:** 2007-08-01

**Authors:** Su Jung Yang

**Affiliations:** 1 Department of Microbiology, Oregon State University, Corvallis, Oregon 97331-3804, USA

## Abstract

Vaccinia virus (VV) undergoes a proteolytic processing to evolve from immature virus particles into intracellular mature virus particles. Most of structural core protein precursors such as p4a, p4b, and p25K are assembled into previrions and then proteolytically processed to yield core proteins, 4a, 4b, and 25 K, which become components of a mature virus particle. These structural rearrangements take place at a conserved cleavage motif, Ala-Gly-X (where X is any amino acid) and catalyzed by a VV encoded proteinase, the I7L gene product. The VV A12L gene product, a 25 kDa protein synthesized at late times during infection is cleaved at an N-terminal AG/A site, resulting in a 17 kDa cleavage product. However, due to the distinct characteristics of A12L proteolysis such as the localization of both the A12L full-length protein and its cleavage product in mature virions and two putative cleavage sites (Ala-Gly-Lys) located at internal and C-terminal region of A12L ORF, it was of interest to examine the A12L proteolysis for better understanding of regulation and function of VV proteolysis. Here, we attempted to examine the *in vivo *A12L processing by: determining the kinetics of the A12L proteolysis, the responsible viral protease, and the function of the A12L protein and its cleavage events. Surprisingly, the A12L precursor was cleaved into multiple peptides not only at an N-terminal AG/A but also at both an N- and a C-terminus. Despite the involvement of I7L proteinase for A12L proteolysis, its incomplete processing with slow kinetics and additional cleavages not at the two AG/K sites demonstrate unique regulation of VV proteolysis. An immunoprecipitation experiment in concert with N-terminal sequencing analyses and mass spectrometry led to the identification of VV core and membrane proteins, which may be associated with the A12L protein and suggested possible involvement of A12L protein and its cleavage products in multiple stages in virus morphogenesis.

## Background

Vaccinia virus (VV), the prototype member of the *Poxviridae *family has a large double-stranded DNA genome. Replication and viral assembly occur entirely in the cytoplasm of host cells, in particular, in areas referred as viroplasms or virosomes. Virus assembly initiates at virosomes surrounded by crescent membranes, which subsequently engulf granular materials forming spherical-shaped particles named immature virions (IV). The IVs transform into brick-shaped structures referred to as intracellular mature virions (IMV) where viral DNAs become condensed and packaged in an electron dense area and are covered by a viral envelope membrane. A portion of IMVs is enwrapped by a membrane cisternae derived from the *trans*-Golgi network and results in the formation of intracellular enveloped virus (IEV), which then becomes fused with the plasma membrane. If the IEVs remain associated with the cells, they are referred to as cell-associated enveloped virus (CEV), or if the IEVs bud through the plasma membrane spreading outside of the cells, they are considered extracellular enveloped virus (EEV).

Despite intensive study of VV morphogenesis, the mechanism required for the transformation of IV to IMV still remains poorly understood. The complex morphological development during the transition initiates with successful DNA replication, concatermer resolution [1,2] and condensation/packaging of the viral genome in IV particles [3]. This is followed by encapsidation of a transcription complex, formation of a defined core, and reorganization of virion membranes [4]. In order to complete this morphogenic transformation, VV undergoes a various post-translational modifications such as proteolytic processing of VV structural proteins, which contributes to proper virus morphogenic development and acquisition of viral infectivity.

**Figure 1:**
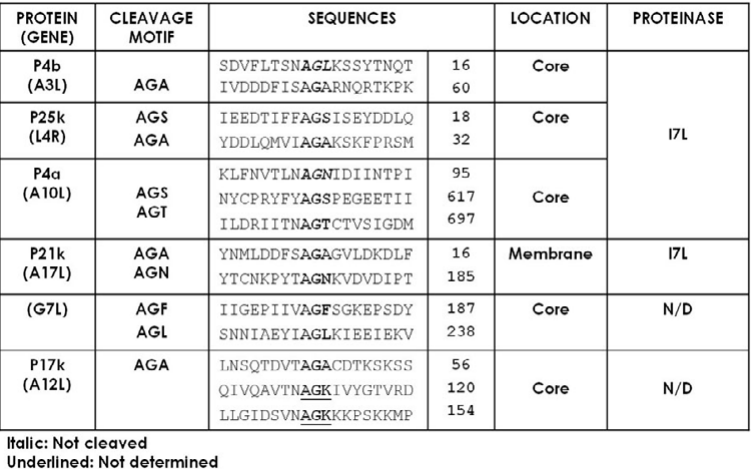
**Vaccinia virus morphogenic proteolysis**. VV has six structural precursor proteins, which undergo morphogenic proteolysis. The consensus motif is not enough to induce VV proteolysis. From left to right, the figure shows the name of gene products, their cleavage motif (italic: not utilized, underlined: not determined), the localization of cleavage product, and the responsible proteinase.

The cleavage processing of VV structural precursor proteins are well studied. The cleavage reactions take place after the second Gly residue of an Ala-Gly-X (AG/X) conserved motif, as indicated in Figure 1. Most precursor proteins show acidic upstream and basic downstream charge differential across the cleavage site, which are usually located within the N-terminal 60 amino acid residues and catalyzed by I7L, a cysteine proteinase [5]. As an example, p4b (A3L) and p25K (L4R) are synthesized at a late stage in the virus life cycle with molecular weights of 66 kDa and 28 kDa, and are proteolytically processed at an N-terminal AG/A site to yield a 60 kDa peptide, 4b and a 25 kDa cleavage product, 25 K respectively [6]. P4a, however, a 102 kDa precursor protein undergoes cleavage events at two different AG/X motifs: an AG/S and an AG/T located at amino acids 619 and 697 [7,8]. Proteolysis at the AG/S and the AG/T sites leads to the release of a 62 kDa (4a) and a 23 kDa C-terminal peptide. Cleavage at the N-terminal AG/A site in A17L processes a 23 kDa full-length precursor protein (p21K) into a 21 kDa peptide (21 K) and additional cleavage at the C-terminal AG/N site is catalyzed by the I7L core protein proteinase [9]. G7L also utilizes two distinct motifs, AG/F and AG/L. Mutagenesis studies have demonstrated that both of these sites are essential for the production of infectious virus [10]. Although a partial cleavage was observed at an AG/S motif in the p25K ORF with an larger molecular weight of 25K, referred as 25K' (Fig. 1), the tripeptides such as AG/L and AG/N located in the N-terminus of p4b and p4a ORF do not serve as reaction sites. These alternate sites, however, do appear to be utilized for the proteolysis of G7L and A17L. Thus, it is of interest to note that the presence of the consensus cleavage motif is not sufficient enough to induce VV proteolysis. Rather, the proteins destined for VV AG/X cleavages are 1) late gene products, 2) catalyzed by I7L proteinase, and 3) incorporated within the core of assembling virions. These represent the characteristics of VV morphogenic proteolysis, which requires a contextually constrained regulation.

The VV A12L protein is synthesized at a late stage with an apparent molecular weight of 25 kDa (p17K) and is proteolytically processed at an N-terminal AG/A site yielding a 17 kDa polypeptide (17 K) similar to p4b and p25K. However, unlike the core protein precursors, of which only the processed forms, 4b and 25 K, are localized to the mature virion, both p17K and 17 K are observed in the core of mature virus, indicating distinct regulation/function of VV proteolysis [11]. In addition, A12L contains two other AG/K sites in the internal region and C-terminus of A12L open reading frame (ORF), of which utilization for VV cleavage events has not been reported. Thus, the research on A12L proteolytic processing may contribute to the discovery of requirements to initiate and regulate viral cleavage processing other than the consensus of cleavage residues, identification of novel AG/X cleavage motif, and elucidation of more detailed function of VV proteolysis in the morphogenic transition. Here, we attempted to characterize the proteolytic processing of the A12L protein through determination of the kinetics, the sites selected for the cleavage reactions, and identification of the responsible protease. We also sought to demonstrate possible A12L associations with other VV proteins, providing a clue to the biological function of the A12L proteolysis in virus assembly.

## Results

### Multiple cleavage products of A12L protein *in vivo*

**Figure 2:**
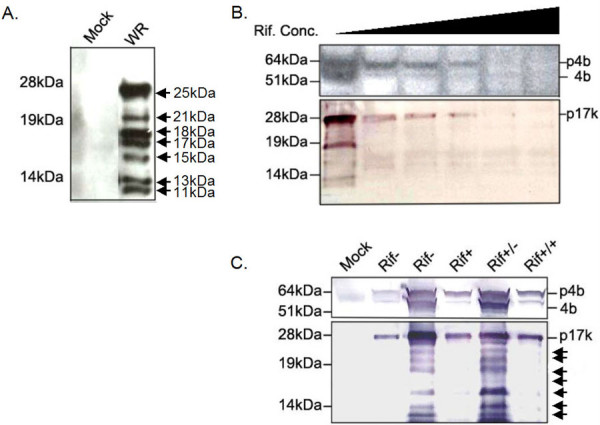
**Multiple cleavage products of A12L protein**. A. BSC-40 cells were infected by VV WR and harvested at 24 hpi. Mock: cells alone, WR: VV WR-infected cell extracts. B. In order to determine whether A12L undergoes proteolysis, BSC-40 cells were infected with VV WR for 24 hours and incubated with rifampicin at concentrations of 0, 100, 150, 200, 300, and 400 μg/ml from left to right. As a positive control of drug induced-inhibition of VV proteolysis, p4b processing was demonstrated. C. Rifampicin-reversibility experiment. Cells were infected with VV and treated with rifampicin (150 μg/ml) at 5 hpi. The rifampicin was replaced with new infection media with and without the drug at 19 hpi to determine the effects of the drug on A12L protein processing for 12 hours. Mock (lane1): cells alone, Rif- (lane 2): rifampicin-free cell extracts harvested at 5 hpi, Rif- (lane 3): rifampicin-free cell extracts harvested at 19 hpi, Rif+ (lane 4): rifampicin-treated cell extracts harvested at 19hpi, Rif+/- (lane 5): rifampicin treated cells at 5 hpi and placed with new media without the drug at 19 hpi, Rif+/+ (lane 6): rifampicin treated cells at 5 hpi and replaced new media containing rifampicin at 19 hpi. Both Rif+/- and Rif+/+ were harvested at 31 hpi.

Previous work by Whitehead and Hruby [11] demonstrated that both the A12L precursor, p17K, and the AG/A cleavage product, 17 K, were present in the core of assembling virions. To determine if any other A12L-derived protein species were evident within the cytoplasm of VV-infected cells, cytoplasmic extracts were prepared and subjected to immunoblot analysis using A12L antisera (anti-A12L) directed against the entire A12L protein. Surprisingly, not only were the 25 kDa (p17K) and the 17 kDa (17 K) proteins detected, but also five other peptides with apparent molecular weights of 21, 18, 15, 13 and 11 kDa were observed (Fig. 2A). Pre-immune sera of A12L did not cross-react with any of these peptides, suggesting that all of the proteins are indeed A12L-derived products (data not shown).

In order to determine if VV proteolysis produces a number of A12L-derived peptides, we compared the pattern of A12L maturation processing in the presence and absence of rifampicin (Rif). Rifampicin, an antibiotic, is known to reversibly block the assembly of VV by disrupting the viral membrane biogenesis and arresting maturational events of the structural core proteins, such as p4a and p4b [12]. Thus, rifampicin has been used to determine the relationship of VV precursor proteins and cleavage products. VV-infected cells were incubated with rifampicin at various concentrations from 100 to 400 μg/ml for 24 hours (Fig. 2B). Using p4b as a positive control, we were able to show the suppressed cleavage at concentrations of 100~200 μg/ml of rifampicin, while proteolysis was observed only in the absence of rifampicin. Drug concentrations of more than 200 μg/ml inhibited the expression of both precursor proteins, p4b and p17K. Similar to p4b processing, p17K was expressed in the presence and absence of rifampicin, whereas the smaller peptides were produced only in the absence of the drug, indicating that p17K is processed into multiple peptides by VV proteolytic processing. Next, we performed a rifampicin-reversibility experiment to confirm that the A12L proteolytic processing is regulated by rifampicin (Fig. 2C). The hypothesis that the rifampicin-arrested proteolysis of A12L would be re-initiated by the removal of the drug was proposed from the previous core protein processing experiments. Infected cells were treated with rifampicin at 5 hpi to allow sufficient A12L precursors to be expressed, and incubated for the next 14 hours to suppress VV proteolysis. Rifampicin-induced suppression of VV cleavage processing resulted in no production of the A12L-derived peptides (Fig. 2C, lane 4). The removal of rifampicin, however, displayed the A12L-derived multiple cleavage products whereas the continuous presence of rifampicin completely suppressed the proteolysis of A12L (Fig. 2C, lane 5 and 6), indicating a rifampicin-regulated A12L proteolysis. In order to rule out the possibility of protein degradation, all the cell lysates were resuspended in PBS with a protein inhibitor cocktail tablet and the same amount of proteins were loaded for the immunoblot analysis. Thus, it is concluded that the A12L protein is proteolytically processed into six peptides, including 17 K, in a similar morphogenesis-associated manner to other VV core proteins.

### Kinetic analysis of A12L

**Figure 3:**
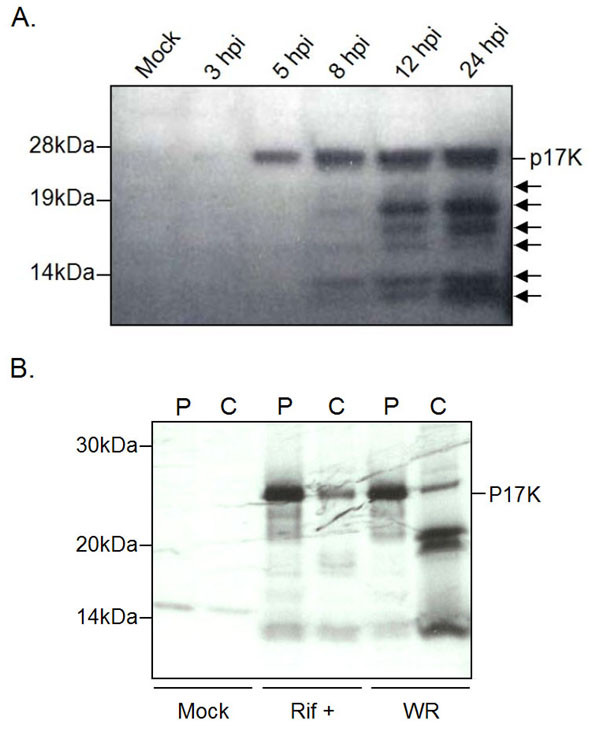
**Kinetic analysis and pulse chase of A12L protein**. A. To determine kinetic analysis of proteolytic processing of A12L, BSC-40 cells were infected with VV WR synchronously and harvested at different time courses as indicated above each lane. A 25 kDa protein corresponds to the A12L precursor (p17K), while smaller peptides with the molecular weights from 21 to 11 kDa are suspected to be the A12L cleavage products. B. Immunoprecipitation of pulse-chase labeled VV-infected cell extracts. Infected cells were labeled with [^35 ^S]-methionine for an hour at 5 hpi and chased with 100× non-radioactive methionine/cysteine. Each pulse (P) and chase (C) of cells alone (Mock), rifampicin-treated (Rif+), and WR infected cell extracts (WR) were analyzed.

For the kinetic analysis of A12L protein processing, cell extracts were prepared at various times post infection and equal amounts of the cell lysates were loaded for the immunoblot analysis (Fig. 3A). The 25 kDa precursor of A12L (p17K) was first detected at 5 hours post infection (hpi), demonstrating that the A12L protein is a late gene product. Over time the amount of the 25 kDa species accumulated throughout from 5 to 24 hpi. The 18, 15, 13, and 11 kDa bands were first detected at 8 hpi and accumulated from 8 to 24 hpi whereas the 21 and 17 kDa peptides began to appear at 12 till 24 hpi. Although the A12L full-length protein is being expressed at 5 hpi, its processing appears to be initiated at 8 hpi and reaches a steady-state at 12 to 24 hpi. This is albeit slow compared to the processing of other core proteins, which are completed within 4 to 6 hpi [7]. The slow kinetics of the A12L cleavage event may be attributed to the possibilities of either inefficient processing or different regulation of the A12L proteolysis from other major core precursors. Moreover, the total numbers of cleavage products imply other possible cleavage reactions, occurring not only at the AG/A site, but also at other residues such as the two AGK sites.

To examine further characteristics of A12L processing, a pulse-chase labeling experiment was conducted in concert with immunoprecipitation (Fig. 3B). Using cells alone as a negative control, we were able to demonstrate that the full-length A12L protein was chased into four peptides with apparent molecular weights of 25, 21, 17, and 11 kDa. P17K remained relatively faint while the 21, 17, and 11 kDa species became more evident after 19 hours of chase. The absence of these four peptides in the rifampicin-treated cells confirmed that all of these peptides are cleavage products. Importantly, the precursor remained after the chase suggests that the cleavage reaction of the A12L protein did not proceed to completion. Rather, the proteolysis of A12L was halted when a steady-state mixture of intermediates was obtained. This could be explained by the fact that the full-length protein by itself may be required for assembling of mature virions or once the quantitative requirement of the intermediate and final peptides is met, the A12L proteolytic processing may be arrested.

### Predicted characterization of A12L proteolysis

**Figure 4:**
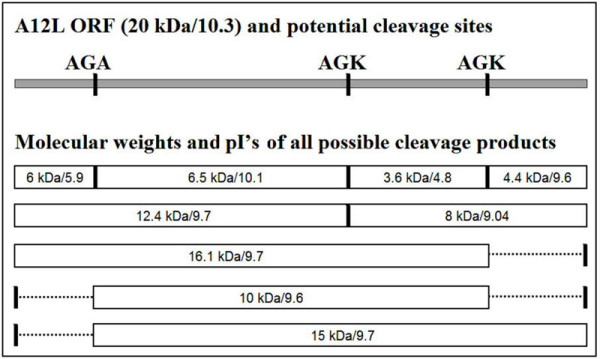
**The predicted molecular weights and pI's of potential A12L cleavage products**. The schematic cleavage products at each AG/X site were drawn with the molecular weights of 6, 6.5, 3.6, and 4.4 kDa. Utilizing single and double AG/X sites, proteolytic processing of A12L were predicted as follows: cleavage at the middle AG/K site would only produce a 12 kDa and a 8 kDa peptide, while cleavages at the C-terminus AG/K site and the N-terminus AG/A site only would introduce a 16 kDa and a 15 kDa product (bottom), respectively. The utilization of both AG/A and N-terminal AG/K site would generate a 10 kDa peptide.

Due to the multiple cleavage products, their molecular sizes, and the slow kinetics, it was of interest to determine the cryptic proteolysis events at AG/K sites. The sequences of A12L proteins encoded by several representative orthopoxviruses show a highly conserved alignment (>95% identity), indicating that A12L may be essential for virus replication. Moreover, both the N-terminal AG/A, and the two AG/K motifs are conserved, suggesting that these motifs are possibly required for maintaining protein function and performing the cleavage reaction properly. As an attempt to identify the cleavage motifs, we considered the possible schematic cleavage products by utilizing different combinations of all three AG/X sites. The relative position of the three AG/X motifs within the A12L ORF is shown in Figure 4. The molecular sizes of the predicted cleavage products and their calculated isoelectric points (pI's) for both complete and incomplete processing of the A12L precursor are also indicated. If all three sites were utilized and the processing proceeds to completion, four small proteins with molecular weights of 6.5, 6, 4.4, and 3.6 kDa would be produced. On the other hand, single site utilization would produce only one or two major fragments with molecular weights of 15, 12.4, 8, and 16 kDa. Thus, the total six A12L cleavage products and their molecular sizes from 11 to 21 kDa suggest that A12L proteolysis may partially take place at all of the AG/X sites, and some peptides are subject to following cleavage reactions. However, due to the discrepancy observed between a predicted and an apparent molecular weight of A12L full-length protein, it was hard to figure out the AG/X serving residues for the proteolysis.

Of note, for the three major core protein precursors, p4a, p4b, and p25K, the portion of the protein that is removed by proteolysis is acidic (pI's of 4.04, 4.08, and 3.26, respectively). Among the potential A12L fragments, only the 6 kDa (pI 5.9) and the 3.6 kDa (pI 4.8) have similar characteristics. Since the 6 kDa protein is not detected after 17 K production, the 3.6 kDa peptide might be designed to be cleaved off. This implies that the AG/K residues may serve as a cleavage motif for A12L fragmentation.

### AG/A utilization and C-terminal proteolysis

**Figure 5:**
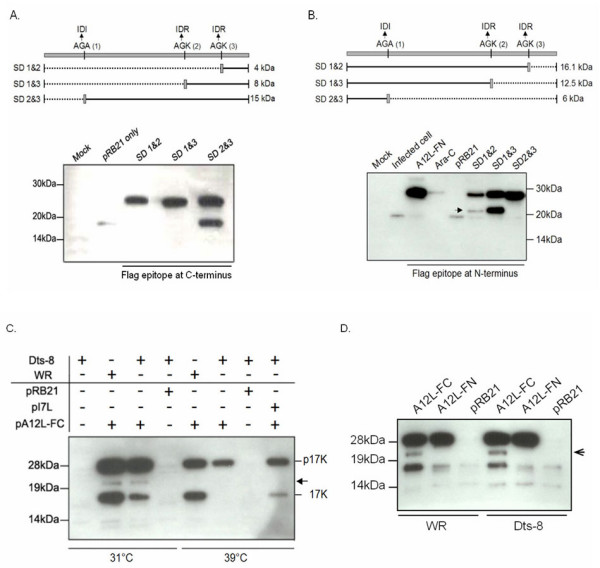
**Proteolysis of A12L**. In order to characterize the proteolytic processing of A12L, we examined the utilization of each AG/X site and determined the responsible proteinase for the processing. A. The A12L ORF with double AG/X site mutations were placed into pRB21 and appended with a C-terminal FLAG epitope (FC). The N-terminal AG/A site and internal AG/K site mutations, the N-terminal AG/A and C-terminal AG/K site mutations, and the internal and C-terminal AG/K site mutations were indicated as SD 1&2, SD 1&3, and SD 2&3, respectively. Each transient expression would result in 4, 8, and 15 kDa cleavage product by cleavages at the C-terminal and internal AG/K residues, and N-terminal AG/A site. B. All of the plasmids contained the same mutations as described above except a FLAG epitope in the N-terminus (FN) of A12L ORF. Ara-C refers to the cells transfected with pA12L-FN in the presence of cytosine arabinoside (Ara-C, 40 μg/mL) as an inhibitor of VV late gene expression. The FLAG tag at the N-terminus of each mutant plasmid would represent the products of 16, 12, and 6 kDa peptides resulted from utilization of the C-terminal, internal AG/K, and N-terminal AG/A site. pA12L-FN: A12L intact ORF under an early/late synthetic promoter. An Arrow indicates a cleavage product near N-terminus. C. BSC-40 cells were transfected with a plasmid containing a FLAG epitope at C-terminus of A12L ORF (pA12L-FC) and infected with WR or Dts-8 (I7L temperature-sensitive mutant virus). Having WR-infected cells as a positive control, Dts-8 infection at the permissive (31°C) and non-permissive (39°C) temperatures showed I7L participation in A12L cleavage event. pRB21: vector plasmid containing an early/late synthetic promoter. pI7L: plasmid born I7L in pRB21. D. To determine another cleavage reaction at N-terminus as indicated with arrow at Fig. 5C, the pA12L-FC and pA12L-FN were transfected into BSC-40 cells and infected with VV WR and Dts-8 at an MOI of 5 PFU/cell. Both infections were incubated at permissive temperature.

In order to demonstrate the utilization of each AG/X site in the A12L ORF, we constructed A12L expression plasmids, which contained AG/A and AG/K site mutations into ID/I and ID/R, respectively (Fig. 5A). In addition, a FLAG epitope was attached at the C-terminus of the A12L ORF to discriminate the mutated plasmid expression from the wild-type endogenous protein processing. To examine the capability of a single site as a cleavage residue, different combinations of two sites were chosen as follows; N-terminal AG/A and middle AG/K site-directed mutations (SD1&2), N-terminal AG/A and C-terminal AG/K site-directed mutations (SD1&3), and middle and C-terminal AG/K site-directed mutations (SD2&3). Under the assumption that each AG/X site is being utilized, there would be peptides corresponding to the sizes of 15, 8, and 4 kDa, resulting from N-terminal AG/A, middle AG/K and C-terminal AG/K cleavages respectively. Although all of the A12L constructs with double mutations demonstrated the full-length proteins, only the SD2&3 plasmid showed the signals corresponding to a 17 K. This result directly demonstrated a cleavage event only at the AG/A site without the utilization of AG/K residues. Similarly, N-terminal fragments produced by each cleavage at C-terminal AG/K (SD1&2), middle AG/K (SD1&3), and N-terminal AG/A (SD2&3) would be 16, 12.5, and 6 kDa in size, respectively (Fig. 5B). None of the A12L mutant constructs conjugated with a FLAG epitope at the N-terminus displayed a 17 kDa AG/A cleavage product due to the loss of N-terminal signal. Instead, the N-terminal AG/A site mutated A12L constructs such as SD1&2 and SD1&3 introduced a 21 kDa peptide (Fig. 5B, arrow), which is attributed to possible proteolysis between C-terminal AG/K and the end of C-terminus. The absence of a 21 kDa signal in intact A12L with a FLAG at the N-terminus (pA12L-FN) may be explained by the complete AG/A site cleavage prior to the C-terminal processing while the absence of a FLAG signal by the SD2&3 plasmid transfection is possibly due to degradation of N-terminal residues as previously observed.

Here, we were able to report only the AG/A site selection as an active cleavage residue, ruling out the possibility of AG/K site utilization. Instead, possible proteolysis was observed to take place at the C-terminus, yielding a 21 kDa species. These were confirmed by the transient experiments of single site mutated A12L with FLAG tag at C- and N-terminus (data not shown). Only the AG/A site mutated A12L with a FLAG tag conjugated at the C-terminus failed to demonstrate a 17 K while the same site mutated A12L plasmid with a FLAG tag appended at N-terminus displayed a 21 kDa peptide. In addition, we were not able to detect the other A12L cleavage products in this transient experiment. Possible reasons are that cleavage events, which occur near the C- or N-terminus would result in the degradation of FLAG-tagged small peptides, or the FLAG epitope interrupts protein folding, allowing only partial cleavage. More likely, the cleavage reactions occur in a cascade. If proteolysis takes place first at an AG/A site, followed by another cleavage in close proximity to the C-terminus, a FLAG epitope at either end of A12L ORF would not detect any further cleavage products.

### AG/A site cleavage by I7L, the VV proteinase

Since its maturation showed similar characteristics as p25K and p4b, whose cleavages are driven by the VV I7L cysteine proteinase, it was likely that A12L might be another substrate of I7L. By taking advantage of a temperature-sensitive mutant virus of I7L, named Dts-8 [13], we were able to compare the processing of transiently expressed A12L protein with a FLAG epitope at its C-terminus (pA12L-FC, Fig. 5C). While the full-length protein and 17 K species were observed at the permissive temperature (31°C), the 17 K species were absent at the non-permissive temperature (39°C), suggesting that I7L is the protease responsible for the AG/A cleavage of A12L. This result was confirmed by a rescue experiment using plasmid borne I7L (pI7L), which permitted p17K to be processed into 17 K at the non-permissive temperature. Using as a plasmid vector alone, pRB21 as a negative control, we did not see any signal under the permissive and non-permissive temperatures, indicating the signals are FLAG-specific. Consequently, we concluded that the I7L protease is responsible for an AG/A site cleavage reaction. However, it has not been determined whether I7L protein participates in the production of the peptides other than a 17 K.

### Priority of N-terminal cleavage of A12L

The transient expression of the A12L with a FLAG epitope and pI7L showed not only a 17 K but also some faint signal at the approximate molecular weight of 21 kDa (Fig. 5C, arrow). In order to determine if a 21 kDa species is not Dts-8 virus specific or non-specific FLAG signal but another cleavage product of A12L protein, we repeated the transient experiment of pA12L-FC and pA12L-FN, followed by WR and Dts-8 infection at the permissive temperature. As shown in Figure 5D, both WR and Dts-8 infection demonstrated a 17 K and a 21 kDa species in the expression of pA12L-FC. However, none of the cleavage products appeared in the expression of pA12L-FN. This indicates that a 21 kDa peptide is not non-specific FLAG signal but an A12L fragment processed near N-terminal end. The relatively weak intensity of 21 kDa species suggests that it might exist as an intermediate cleavage peptide rather than a final product. Taken together with the fact that a FLAG tag at an N-terminus of A12L did not show any band, A12L proteolysis events are expected to occur at an N-terminus and then followed by a C-terminal proteolysis.

### Intracellular localization of A12L and its cleavage products

**Figure 6:**
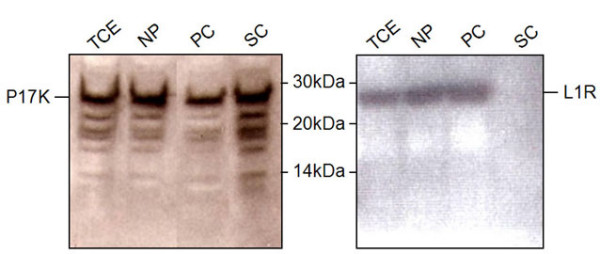
**Subcellular localization of A12L protein**. BSC-40 cells were infected with WR at an MOI of 10 PFU/cell and the cell extracts were separated by differential centrifugations. TCE: total cell extracts, NP: nuclear pellet fraction, PC: particulate cytosolic fraction, SC: soluble cytosolic fraction. Right and left panels show the localization of A12L and L1R proteins.

Since an N-terminal AG/A cleavage is observed in the A12L protein, it was hypothesized that the removal of N-terminal residues might be required for the proper localization of A12L-derived peptides. Other core proteins such as p25K (L4R) have been shown to be cleaved at an N-terminal AG/A site like A12L protein. Failure of this cleavage in p25K resulted in impaired intraviral localization and loss of packaging into virions. [14] This is commonly observed among different viruses, which express polypeptides and localize their cleavage products into different subcellular locations. Thus, we attempted to determine whether the AG/A cleavage of A12L results in different intracellular localization of the cleavage products from the precursor. The infected cell lysates were fractionated by differential centrifugation to yield a nuclear pellet fraction (NP), a particulate cytosolic fraction (PC), which includes whole virions and membraneous components, and a soluble cytosolic fraction (SC). As a control, the subcellular localization of the L1R gene product was examined (Fig. 6). The L1R gene product, a VV membrane protein, is known to be located in the nucleic and the membraneous fraction but not in the soluble cytosolic fraction [15]. The distribution of L1R demonstrated the differential centrifugation was conducted properly. Both A12L full-length protein and its cleaved peptides were localized to not only nuclear pellet fractions but also soluble/particulate cytosolic fractions of the total lysates. This implies that the cleavage at an AG/A site in the A12L ORF does not lead to different subcellular localization of the cleavage products. Rather, the full-length proteins distributed all around the cytoplasm undergo proteolytic processing, generating multiple peptides, which are not re-located into the virion-containing fraction. It is an indicative of the unique characteristics of A12L proteolysis not subjected to the contextual processing, which refers to as a cleavage reaction occurred within the context of assembling mature virions [16].

### Possible association of A12L with a variety of VV proteins

**Figure 7:**
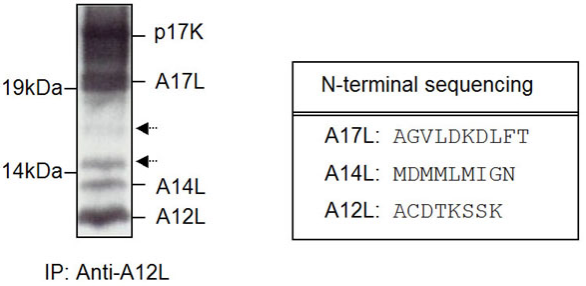
**Identification of A12L-derived peptides**. BSC-40 cells were infected with WR at an MOI of 5 PFU/cell, of which cell lysates were subjected to immunoprecipitation analyses with anti-A12L. The immunoprecipitates were resolved in 12% gel, transferred to PVDF membrane, followed by Coomassie staining. The four bands in molecular weights of 20, 15, 13, and 11 kDa were cut out and sent for N-terminal sequencing. The sequence data we obtained from N-terminal sequencing is represented in the table below. Arrows indicate the peptides, which are N-terminally blocked or not enough protein to analyze the amino acid sequences.

In order to identify the cleavage residues of the A12L-derived peptides, immunoprecipitation of A12L was performed and resolved on 12% NuPAGE Bis-Tris gel electrophoresis. Figure 7 shows the PVDF membrane, which A12L immunoprecipitates were transferred onto and stained with Commassie R-250. Five bands were detected with approximate molecular weights of 21, 17, 15, 13, and 11 kDa. Surprisingly, only one of the four peptides corresponding to 11 kDa turned out to be A12L, which was cleaved at an N-terminal AG/A site. In contrast, the ~21 kDa peptide was identified as an A17L gene product, a virion membrane protein while the 13 kDa peptide matched with the A14L protein. The sequence of the 21 kDa peptide represents a cleavage product (21 K) of the 23 kDa full-length A17L protein (p21K), being generated by the removal of the N-terminal 16 amino acids. The cleavage product of A17L, a 21 K is previously reported to interact with the gene product of A14L, a phosphorylated membrane protein and induce the initial sequence of events of VV membrane formation [17,18]. Although we were able to obtain sequence of each of the three peptides, some of them were mixed with other protein sequences and not enough protein of the 17 and 15 kDa (as indicated with arrows at Fig. 7) was obtained for N-terminal sequencing analysis. Thus, to identify other cleavage residues and determine more clearly which viral proteins A12L protein incorporates with, we loaded the A12L immunoprecipitates on 2-dimensional (2D) PAGE gel for better resolution, analyzed them through N-terminal sequencing analysis and mass-spectrometry for acquisition of protein sequences.

**Figure 8:**
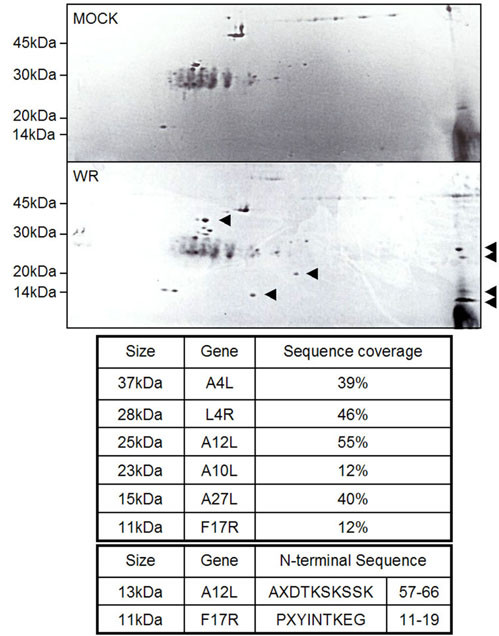
**Possible association of A12L with other VV proteins**. The anti-A12L immunoprecipitates were absorbed in IPG strips for two dimensional gel eletrophoresis (2D-gel), which were stained with Coomassie R-250. The distinguished spots were cut out and sent for either N-terminal sequencing or MS analyses (LC-ESI-Q-TOF MS). The upper panel shows the immunoprecipitates of the cells alone (Mock) while the bottom panel is WR-infected cell lysates (WR) immunoprecipitated with A12L antibody. Arrowheads are the A12L-derived peptides distinguished from mock (upper panel) and antibody alone (data not shown). The table underneath the 2D gel stains shows the summary of the total results from both analyses.

Compared to a negative control, mock (Fig. 8) and antibody of A12L alone (data not shown), A12L specific peptides were separated into six different sizes; 37, 28, 25, 23, 15, 13, and 11 kDa. Through the N-terminal sequencing analysis (Fig. 8 bottom panel), we identified a 13 kDa peptide as an A12L gene product, which contains the amino acids (aa) of 57 to 66 residues and a 11 kDa peptide as a F17R gene product with amino acid sequences from 11 to 19 residues, which were mixed with the same sequences as the 13 kDa A12L peptide. Due to N-terminal blockage of the other peptides, we employed mass spectrometry to identify the proteins. As a result, a variety of different VV proteins with sequence coverage from 12 to 55% were obtained, which is above the minimum coverage (5%) for protein identification. The A12L-immunoprecipitates with the molecular weights of 37, 28, 25, 23, 15, 13, and 11 kDa turned out to be a gene product of A4L, L4R, A12L (full-length), A10L, A27L, A12L (cleaved at AG/A) and F17R, respectively (Fig. 8). It is interesting to report that the A12L immunoprecipitates turned out to be VV core (A4L, A10L, L4R, and F17R) and membrane (A17L, A14L, A27L) proteins. The gene product of A4L, a 39 kDa core protein, associates with a 60 kDa cleavage product (4a) of A10L, and stimulates proper progression of IV to IMV [19,20] as two other core proteins, L4R and F17R, are participated in correct viral genome packaging, which is an essential step for assembling mature virions. On the other hand, A27L, a 15 kDa VV envelope protein also incorporates with A17L just like A14L, and responsible for envelopment of IMV particles [17,21,18]. Therefore, the A12L protein with these viral associates may imply its possible participations in different stages during VV morphogenic transitions.

## Discussion

Investigation of the proteolytic maturation of the VV A12L core protein yielded several unexpected results. It is most interesting that proteolytic processing of the VV A12L protein produces a mixture of products and does not proceed to completion, as do the other VV core proteins. There are two hypotheses to consider for this phenomenon. First, perhaps some of the multiple cleavages are "accidental", occurring due to a quirk of having cryptic AG/X sites within the precursor. This assumption appears unlikely since all of the sites are well conserved with the orthopoxviruses and the viruses have had ample time to remove the sites by mutation if cleavage was deleterious. Furthermore, other core protein precursors have cryptic cleavage sites, (AG/S in p25K, and AG/N in p4a) which are either not recognized or do not interfere with the reaction proceeding to completion. Second, a more intriguing possibility is that the incomplete processing of the A12L precursor is required to produce multiple protein species, some of which might have different functions. Certainly for other viruses such as poliovirus, partially cleaved peptides are known to have different functions from the fully maturated products [22]. In addition, the A12L proteolysis not in context with assembly of mature virions suggests that both of A12L precursor and cleavage fragments may play dual roles as structural components of mature virion and as non-structural proteins.

In contrast to the presence of multiple cleavage products *in vivo*, only AG/A site cleavage is reported here, catalyzed by the I7L VV core protein proteinase. Despite no observation of cleavage at the putative AG/K residues, it cannot be ruled out that the AG/K sites may become recognizable by the proteinase after the first cleavage. In consideration of the fact that the A12L proteolysis takes place at an N-terminus in advance to a C-terminal cleavage, it is more convincing to speculate that the A12L cleavage is regulated in order, so that a blockage of cleavage reaction may inhibit subsequent cleavage processing by forming an improper structure, which is not fully accessible to the proteinase. The proteolysis at both ends of A12L ORF, however, raises another possibility of cleavage reactions at a new motif other than the AG/X sites in concert with involvement of another proteinase. Given this atypical behavior, it is of interest to determine the essentiality of the A12L protein in viral replication. Therefore, a conditional A12L mutant virus may need to be designed and used to address the role of A12L as well as how important each AG/X site is to the function of A12L.

The identification of the numbers of viral proteins immunoprecipitated with A12L antibody is contradictory to the fact that A12L precursor proteins are processed into the multiple peptides. This result could be explained by cross-reactivity of A12L antibody. Considering the rifampicin-regulated A12L cleavage processing, it would be likely that the antibody of A12L precipitates virus-encoded late gene products. However, the parallel immunoprecipitation with A17L and F17R antibodies, followed by immunoblot analyses with A12L antibody demonstrated positive signal of A12L from each A17L and F17R immunoprecipitate, (see Additional file 1). This confirms the A12L associations with A17L and F17R proteins and supports the possible association of A12L with A14L and A27L proteins. In case of F17R, the precipitated A12L fragment by F17R antibody has previously demonstrated (personal communication). Thus, it is more likely that A12L may have associations with other viral membrane and core proteins, ruling out the non-specific cross reactivity of A12L antibody. To confirm the association of A12L with the other proteins and determine their biological function, each associate needs to be more characterized.

Recent studies of early morphogenic processing events have provided the participation of the membrane proteins such as A17L, A14L and A27L in early development of IV particles as well as IEV particles, recruiting nascent viral membranes to the viral foci, inducing their stable attachment to the surfaces of viral factories, and developing envelopment of IEV particles [23]. Unlike these membranous proteins, the association of A4L with A10L plays a role in the correct assembly of nucleoprotein complex and organization of IV content with the membranes while F17R (a DNA-binding phosphoprotein), and L4R (a DNA-binding protein) are proposed to work for the correct viral genome packaging and efficient transcription [20,24-26]. These participations of the A12L-associated proteins throughout the progression of IV to IMV and IEV particles suggest that the A12L may also be involved in multiple stages of virus morphogenesis.

## Conclusion

In conclusion, we were able to demonstrate that A12L undergoes unique proteolysis, which occurs multiple times in order, utilizing both AG/A site and new cleavage residue other than the AG/X motif, not in context of assembling virions, and shows the possible association with various VV proteins. These characteristics imply more extensive participations of VV proteolytic maturation processing not limited to viral morphogenesis. Further investigation on A12L proteolysis and biological function of each A12L cleavage product will elucidate more details of regulation and function of VV proteolysis.

## Methods

### Cell cultures

VV WR (Western Reserve strain) was grown on confluent monolayers of BSC-40 cells maintained in Eagle's minimal essential medium (EMEM, Invitrogen) supplemented with 10% fetal calf serum (FCS, Invitrogen), 2 mM glutamine (Invitrogen), and 10 mM gentamicin sulfate (Invitrogen) at 37°C in a 95% humidified atmosphere containing 5% CO_2_. For infection of WR, BSC-40 cells were maintained in infection media (EMEM) supplemented with 5% FCS, 2 mM glutamine, and 10 mM gentamicin sulfate and were infected at a multiplicity of infection (MOI) as indicated. Infected cells were harvested by centrifugation at 750 × g for 10 min., and resuspended in phosphate buffered saline solution (PBS), which contained a protease inhibitor mix tablet (Roche), followed by three cycles of freezing and thawing to lyse the cells. After a post nuclear spin at 350 × g at 4°C, cell extracts were subjected to immunoblot or immunoprecipitation analyses.

### Rifampicin-reversibility experiment

Rifampicin stock solution (10 mg/ml, Sigma-Aldrich) was prepared in 100% Dimethyl sulfoxide (DMSO) and diluted out with dH_2_O for various concentrations. BSC-40 cells were synchronously infected with VV WR at an MOI of 5 plaque forming units (PFU)/cell and then treated with rifampicin (150 μg/ml). The treatment with rifampicin was performed at 5 hpi for the rifampicin-reversibility experiment. In order to compare the pattern of proteolysis in the absence and presence of the drug, the VV infected cell extracts were harvested when the drug was added and removed. After the removal of rifampicin, new infection media with and without the drug was replaced. Infected cell pellets were re-suspended in PBS, subjected to three cycles of freezing and thawing, and clarified by low speed centrifugation. Immunoblot analysis was performed on 12% NuPAGE Bis-Tris gels (Invitrogen). Antibody of A12L was generated by bacterial expression of A12L full-length protein, which was fused with an N-terminal 7× His tag and affinity purified over a Ni-NTA-agarose column [11].

### Kinetics of A12L processing

Confluent BSC-40 cells were synchronously infected with VV WR at an MOI of 10 PFU/cell. The infected cells were harvested at various time points after infection (5, 8, 12, and 24 hpi) and resuspended in protease inhibitor-containing PBS, followed by a post-nuclear spin as previously described. The same amount of each sample was resolved on a 12% NuPAGE Bis-Tris gel (Invitrogen) prior to immunoblot analysis with A12L antisera and pre-immune serum was used as a control (data not shown).

### Pulse chase

Confluent monolayers of BSC-40 cells were synchronously infected with VV WR at an MOI of 10 PFU/cell. At 5 hpi, [^35^S]-methionine (10 μCi/mL, EasyTag EXPRE^35^S protein labeling mixture, Perkin Elmer Life Science) was added to the infection medium. After 1 hour, the radioactive medium was replaced with the medium containing 100× non-radioactive methionine/cysteine and chased for 19 hours. The infected cell extracts were used for immunoprecipitation and analyzed by electrophoresis on a 12% NuPAGE Bis-Tris gel. The gel was dried and exposed to a film for 72 hours.

### Immunoprecipitation

Protein A-Sepharose beads (Amersham) were prepared according to manufacturer's instructions. Infected cell extracts were lysed and diluted with Radioimmunoprecipitation buffer (RIPA buffer: 50 mM Tris [pH7.4], 1 mM NP-40, 150 mM NaCl, 1 mM EDTA, 0.25% sodium deoxycholate and protease inhibitor cocktail tablets) and pre-cleared for an hour-incubation with re-hydrated beads at 4°C. After a short spin, the supernatant was transferred to a fresh tube and incubated with A12L antibody overnight at 4°C with shaking. Fresh beads were added and incubated for 2–3 hours at the same temperature. The beads were collected by a short centrifugation at 14,000 × g for 40 sec., followed by three cycles of washing with 50% PBS/RIPA buffer and the final re-suspension in 4× sample buffer. After 5 min. of boiling, the samples were analyzed by gel electrophoresis on a 12% NuPAGE Bis-Tris gel.

### Plasmid construction and transfection

To determine the cleavage residues for A12L protein cleavage processing, three possible AG/X sites (AG/A and two AG/Ks) were changed into IDI and IDR, respectively by Quickchange site-directed mutagenesis kit (Stratagene). The open reading frame (ORF) of both the wild-type A12L (pA12L) and the mutated A12L genes were placed into the pRB21 plasmid [27], which has a VV early/late synthetic promoter. Primers for the site mutations were designed as follows: site-directed mutation 1 (SD1) for the first AG/A mutation at the residues 55–57, 5'-CTT AAT TCT CAA ACA GAT GTG ACT ATC GAC ATC TGT GAT ACA AAA TCA AAG AGT TCA-3', site-directed mutation 2 (SD2) for the middle AGK site mutation at the residues 119–121 into IDR, 5'-CAG ATT GTC CAA GCT GTT ACT AAT ATC GAC CGC ATA GTT TAT GGT ACC GTC AGA GAC-3', and site-directed mutation (SD3) for the C-terminal AGK site mutation at the residues 153–155 into IDR, 5'-CTT CTA GGT ATC GAC TCA GTT AAT ATC GAC CGC AAG AAA CCA TCT AAA AAG ATG CCT-3'. Underlined characters indicate the mutation sites. SD1&2, SD1&3, and SD2&3 are double site mutations generated by using each combination of the primers. In addition, a FLAG-epitope was appended to the C-terminus (FC) and N-terminus of each ORF (FN) to discriminate the transient expression from an endogenous protein processing.

For transfection of the plasmids into BSC-40 cells, infection media of EMEM was placed in new eppendorf tubes and mixed with 2 to 10 μg of DNA and 30 μl of a transfection reagent (DMRIE-C, Invitrogen). The mixture was vortexed, placed at room temperature for 20 min. and loaded on 6-well plates of ~100% confluent BSC-40 cells. Each infection of VV WR or Dts-8 (IHD-J derived I7L-termperature sensitive mutant virus, kindly provided by Dr. Rich Condit) was performed with an MOI as indicated. To determine the responsible protease for A12L proteolysis, we have used pA12L-FC under Dts-8 infection and compared the cleavage pattern at permissive (31°C) and non-permissive (39°C) temperatures. For rescue experiment of I7L proteinase activity, we constructed I7L plasmid in the control of an early/late synthetic promoter as described [13].

### Two dimensional gel electrophoresis (2D gel eletrophoresis)

Monolayers of BSC-40 cells in 100 mm plates were infected with VV WR at an MOI of 10 PFU/cell and harvested at 24 hpi for the immunoprecipitation with anti-A12L as described above. The beads after the final spin were resuspended with 180 μl of rehydration buffer (9 M Urea, 4% CHAPS, 50 mM DTT, 2% ampholyte, and Bromophenol blue) for an hour at room temperature with shaking. After a short spin, the rehydration solution was applied into the strip tray where 11 cm IPG Readystrips with a pH range of 3–10 (BioRad) were positioned overnight. The IPG strips were transferred to a Protean IEF tray (BioRad), which was placed to the Protean IEF cell for isoelectro-focusing. For the second dimensional (2D) gel electrophoresis, the IPG strips were treated with sample preparation buffer (0.0625 M Tris [pH 6.8], 5% β-mercaptoethanol, and 2% SDS), followed by treatment with Equilibration buffer (EB) I and II, which contained 200 mg of DTT and 250 mg of Iodoacetamide respectively in 10 mL of EB (6 M Urea, 2% SDS, 0.05 M Tris [pH 8.8], and 20% glycerol). Then, the IPG strips were rinsed with 1× Running buffer and loaded on precast Criterion gels (BioRad) for separation on the basis of molecular weight. The gels were either stained with Coomassie R-250 solution (0.1% Coomassie R-250, 40% MeOH, and 1% Acetic acid [HoAC]) or transferred to PVDF membrane, followed by the Coommassie stain R-250.

### Mass spectrometry of the A12L-immunoprecipitated peptides

The BSC-40 cell extracts infected with VV WR at an MOI of 5 PFU/cell were subjected to immunoprecipitation with anti-A12L as described above. The immunoprecipitates of A12L protein were resolved on 2D gel, followed by staining with Coomassie R-250 and de-staining until protein bands could be easily visualized. Protein bands of interest were excised in as small of piece of gel as possible. The gel slices were then dehydrated with acetonitrile (AcN) and re-hydrated with 50 mM ammonium bicarbonate. This procedure was repeated and the final dehydration was dried under a vacuum. To each tube 10–40 μL of 1 μg/μL Promega trypsin in 10 mM Tris-HCl, pH = 8.0 was added. After the enzyme solution was fully absorbed, the excess trypsin solution was removed and replaced with 40 μL of 10 mM Tris-HCl, pH = 8.0. Each sample was incubated at 37°C for 12–16 hours. The peptides were then extracted from the gel by vortexing with 40–80 μL of 80% AcN/5% TFA. The extraction fluid was placed in a new tube and concentrated to 10–15 μL. The tryptic peptides were injected onto an HPLC system with a C_18 _column system (Jupiter, 0.2 × 10 mm, 300 Å) followed by liquid chromatography electrospray ionization quadrupole ion trap (LC-ESI-QIT) mass spectrometry (Finnigan LCQ). HPLC was performed with a gradient from 90% Buffer A (0.1% TFA in water) to 90% Buffer B (0.01% TFA and 5% water in acetonitrile) over 80 min [28]. The LC-ESI-QIT MS data was converted into Sequest DTA files and searched with the Mascot program. Mascot (Matrix Science, London, UK) software was used for the protein identification. The uninterpreted tandem mass spectral data were searched against the MSDB database, a composite, non-identical protein sequence database built from a number of primary source databases (Matrix Science).

### Differential centrifugation for subcellular fractionation

Confluent BSC-40 cells were infected with VV WR at an MOI of 10 PFU/cell and harvested as described. From 1 mL of total cell lysates, 100 μl was used as total cell extracts while the rest of the lysate was centrifuged at 700 × g for 10 min. to pellet the nuclei. Subsequent centrifugation at 20,000 × g for 30 min of the supernatant separated the soluble cytosolic fraction from the insoluble cytosolic fraction. Each pellet of nuclei and insoluble fraction was resuspended in 900 μl of PBS [15].

## Abbreviations

VV: Vaccinia virus; IV: Immature virus; IMV: Intracellular mature virus; IEV: Intracellular enveloped virus; WR: VV Western Reserve strain; SD: Site-directed mutagenesis; MOI: Multiplicity of infection; Hpi: Hours post infection.

## Competing interests

The author(s) declare that they have no competing interests.

## Supplementary Material

Additional file 1 Parallel immunoprecipitation of each A17L and F17R antiserum followed by A12L antibody immunoblot analyses. The immunoprecipitates (IP) of A17L and F17R antibody were analyzed with immunoblot assay (IB) with each antibody of A17L, F17R, and A12L. Click here for file
