# Supplementary figures and images for: Characterization of vaccinia virus A12L protein proteolysis and its participation in virus assembly
Source: Virol J. 2007 Aug 1;4:78. doi: 10.1186/1743-422X-4-78 (PMC1959187; doi:10.1186/1743-422X-4-78)

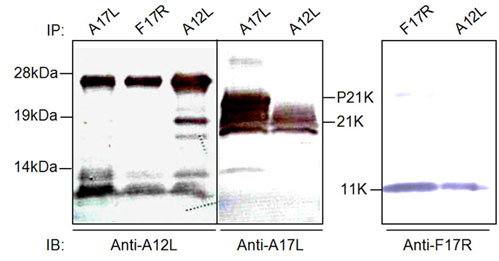

Supplement: Additional file 1 — Parallel immunoprecipitation of each A17L and F17R antiserum followed by A12L antibody immunoblot analyses. The immunoprecipitates (IP) of A17L and F17R antibody were analyzed with immunoblot assay (IB) with each antibody of A17L, F17R, and A12L. [file 1743-422X-4-78-S1.jpeg]
